# Balancing Between Being Proactive and Neutral: School Nurses’ Experiences of Offering Human Papilloma Virus Vaccination to Girls

**DOI:** 10.1177/1059840520933323

**Published:** 2020-06-24

**Authors:** Eva Runngren, Mats Eriksson, Karin Blomberg

**Affiliations:** 1 Faculty of Medicine and Health, School of Health Sciences, Örebro University, Sweden

**Keywords:** human papilloma virus vaccination, school nursing, experience, focus groups, information

## Abstract

The aim of this study was to describe the experiences of Swedish school nurses when they offered the human papilloma virus (HPV) vaccination to girls aged 10–12 years. Four focus groups with a total of 17 school nurses were conducted and analyzed using inductive content analysis. The results showed that the school nurses were balancing between keeping a neutral role and the need to increase the uptake of the HPV vaccination. They described the consent forms and information that they gave the girls and their parents to help them make an informed decision about the vaccination. There were also ethical and moral dilemmas that arose with regard to the HPV vaccinations. Our findings demonstrate the need to provide school nurses with clear guidelines and support, so they can play an active role in interacting with the girls and their parents when they offer the HPV vaccination.

## Background

Cervical cancer is the fourth most frequent cancer form in women worldwide, and figures from the World Health Organization (WHO, 2018) show that this cancer is responsible for more than 270,000 deaths every year. Human papilloma viruses (HPVs), which are a prerequisite to develop cervical cancer, are common all over the world, and most sexually, active males and females will be infected with an HPV at some point in their lives. Most HPV infections are resolved without treatment, but all females face the risk that some HPV infections may progress to cervical cancer over time (Zur Hausen, 2006). Screening for high-risk HPVs provides cost-effective secondary prevention for cervical cancer, when it is combined with the HPV vaccination (WHO, 2018, 2019).

The first HPV vaccine was registered in 2006, and the WHO recommends that all countries should offer it as part of the primary prevention measures included in their national vaccination programs. By March 2017, 71 countries had added the HPV vaccine to their vaccination programs for girls and 11 countries had introduced it for boys (WHO, 2017). One study found that most countries that provide the HPV vaccine do so when the girls are 9–14 years of age (Markowitz et al., 2012). Some countries provide the vaccine in primary care settings, some provide them in schools, and some use both settings (Brotherton & Bloem, 2018). The highest vaccination rates have been achieved by government-financed school programs (Wang et al., 2019).

In Sweden, the HPV vaccine was added to the national vaccination program in 2012, and it is provided free to girls aged 10–12 years by the country’s school health service (Public Health Agency of Sweden, 2018). Boys will be included in the program starting from autumn 2020. The advantage of offering the vaccination through school health services is that all school-aged girls are offered the HPV vaccination and have the opportunity to receive it while undergoing mandatory schooling, until the girls reach an age of 18 years. Depending on the girls’ age and maturity, HPV vaccination can be given without parental consent earlier than 18 years, which is decided on case-by-case basis. In Sweden, the vaccine was administered to girls born in the year 2006 and in 2018, and the uptake was 85% for the first dose and 80% for the second. This is a lower coverage rate than for other vaccinations in the vaccinations program and has not yet reached the goal, which is at least 90% (Public Health Agency of Sweden, 2018).

To make an informed decision about the HPV vaccination, both the girls and their parents need understandable and clear information (Hendry et al., 2013). Research has shown that the type and content of the information school nurses give to them varied. Some provided information that clearly encouraged the parents to vaccinate their child against HPV, while others did not provide adequate information and materials about HPV and the HPV vaccine (Grandahl, Oscarsson, et al., 2014; Hendry et al., 2013).

Schools seem to be the most effective place to let girls and their parents know about the HPV vaccination. School nurses provide an important source of information (Grandahl, Tyden, et al., 2014; Rosen et al., 2016; Wang et al., 2019) and play a significant role in maximizing the coverage of the HPV vaccination.

Altogether, previous studies of HPV vaccination have focused on attitudes and knowledge among different stakeholders (Rosen et al., 2015; Rosen et al., 2018) rather than explore how HPV vaccination is conceptualized and experienced by those who have a key role in the vaccination program. The rationale for this study is based on naturalistic inquiry as a theoretical framework that provides the opportunity to examine subjective and complex experiences in the natural context and setting in which they occurred (Lincoln & Guba, 1985). As reality is seen as multiple, socially constructed, and subjective, we focus here on school nurses’ own subjective experiences to create rich data of the studied phenomena. Therefore, the aim of this study was to describe Swedish school nurses’ experiences of offering HPV vaccinations to girls aged 10–12 years.

## Design and Methods

A qualitative design with focus group (FG) interviews (McLafferty, 2004) was used. Ethical approval for the study was obtained from the Regional Ethical Review Board in Uppsala, Sweden (registration number 2017/243).

### Setting and Participants

The study was carried out in 2017, 5 years after the HPV was added to the Swedish vaccination program. The researcher invited all 50 of the school nurses working in a county in the middle of Sweden that includes both rural and urban areas, to take part in the study. The manager responsible for county’s school nurses helped the researchers to contact the school nurses by forwarding information about the project to them by email. A reminder email with the information was sent once. The email stated that the school nurses could take part if they had at least 2 years’ experience as a school nurse and had offered the HPV vaccination. Of the 50 school nurses who were contacted, 31 did not reply and 2 declined because they were too busy. To our knowledge, there are no differences in working duties between the respondents and no respondents. The 17 regular school nurses who agreed to take part in the study worked in schools situated in areas with a wide range of socioeconomic and ethnic profiles.

### Data Collection

Four FG interviews (McLafferty, 2004) were conducted between June and November 2017, during working hours. The FGs comprised three to six school nurses and were held in locations chosen by the participants: at the university, in one of the schools, in a county council meeting room, and in a café. The FGs were guided by a semi-structured interview format. They explored the school nurses’ experiences of offering the HPV vaccination, the information they provided, and strategies concerning HPV vaccination. Examples of the questions are presented in Table 1. The first FG served as a trial run, but since the basic questions were the same in all groups with the addition of one question for the three subsequent groups, the researchers decided to include the first. The last author (A3), who is a nursing professor with previous experience of conducting FG interviews, was the moderator for the first FG and the first author (A1) was the co-moderator. These roles were reversed for the other three FGs. All the FG interviews were audio recorded and lasted between 45 and 80 min. The interviews were transcribed verbatim by a professional transcription service shortly after the interviews were conducted and were read and checked by the moderator and co-moderator.

**Table 1.** Examples of Focus Group Questions.

How do you look upon your role as a school nurse?
Can you describe your experience of offering the HPV vaccination to girls and their parents?
What information do you give to the girls before the HPV vaccination?
What information do you give to the parents before the HPV vaccination?
What strategies do you use to encourage the girls and their parents to proceed with the HPV vaccination?

Note. HPV = human papilloma virus.

### Data Analysis

All the transcribed interviews were imported into NVivo qualitative data analyses software, Version 11 (QSR International Pty Ltd, Melbourne, Australia). These included the first FG, as it provided rich data. The two researchers (A1 and A3) who performed the FG interviews read all the transcribed interviews to gain an overall understanding of the data that had been collected.

The data were analyzed following a three-phase inductive process devised by Elo et al. (2008), namely, preparation, organization, and reporting. Two of the researchers (A1 and A3) familiarized themselves with the data by reading and re-reading the transcripts and selecting the units of analysis, which were determined by the purpose of the study. These units could either be a word or sentence. The researcher then used open coding to formulate the content of the units, and the codes were grouped together based on their similarities and differences. A process of abstraction was then used to identify the subcategories and one overall category, and these were confirmed by the second author (A2). Once this process had been completed, the research team discussed the preliminary results until they reached a consensus. Individual quotations were identified from the FG to illustrate the findings and increase the trustworthiness of the data (Elo et al., 2008).

## Results

### Balancing the Need to Maintain a Neutral Position but Encourage Uptake

During the analysis, it emerged clear that the school nurses needed to play an active role to encourage vaccination, by providing the girls and their parents with information and actually delivering the vaccine. However, they also felt that they needed to maintain a neutral position, so that they did not unduly influence the decision that the girls and their parents made about whether the vaccine should be provided. Six subcategories were identified. They used strategies to inform the girls and their parents about the HPV vaccination, and this included passing on material provided by official information sources. They maintained a neutral position about the HPV vaccination and dealt with dilemmas that arose about the information that was needed and providing the HPV vaccination. The final two issues were about building a relationship with the girls and their parents that included trust and making sure that the actual vaccination procedure was a positive experience. These six subcategories are presented in Table 2.

**Table 2.** The Main Category With Subcategories Identified in the Focus Group Interviews.

Main category: Balancing the need to maintain a neutral position but encourage uptake
Subcategories
Using strategies to inform the youth and their parents about the HPV vaccination
Perception of official information sources
Maintaining a neutral position on the HPV vaccination
Dealing with dilemmas about information and conducting HPV vaccinations
Building a relationship and trust with the girls and their parents
Making the vaccination a positive experience for the girls

Note. HPV = human papilloma virus.

### Using Strategies to Inform the Youth and Their Parents About the HPV Vaccination

The school nurses said that giving the girls’ parents information about HPV and the HPV vaccination was as essential, but it was also very difficult. The school nurses could either mail the information letters together with a consent form for the HPV vaccination to the parents, or the girls could take the letter home and give it to their parents. After they received the letters, some parents contacted the school nurse and asked for her or his professional point of view about the HPV vaccine. Some of the school nurses said that discussing HPV at parents’ meetings resulted in increased vaccination coverage.

Some of the school nurses talked about the HPV vaccination in school classes, but others said that this didn’t happen in their school. In some cases, only the girls were involved in these briefings, but in other cases, the school nurses talked to the boys and girls together. The school nurses said that it was difficult to know the best way to deliver the information to pupils, as sometimes the students asked lots of questions and sometimes they did not ask any. When they talked to mixed classes, it gave the boys the chance to ask why they did not get the vaccination.

Some of the school nurses said that they did not speak to the girls directly, as sometimes the girls got frightened and stressed when they were reminded about the vaccination. On the other hand, some of the school nurses felt that it was important that the girls were well informed about the HPV vaccine because they could then voice their opinions when their parents were deciding whether to get them vaccinated. One of the school nurses in FG 1 had decided not to hold classroom session with the girls. “This is partly because I think they get so much information anyway and if they are interested, they come to me,” she or he said. The written information that was sent home was standardized and believed to contain sufficient information.

It is not possible to vaccinate the girls if the consent form is not returned to the school nurse, and some of the school nurses said they spent considerable time and effort trying to get the parents to provide consent. Others said that the parents at their school responded more easily. Some asked the classroom teachers to include reminders in the weekly letter they sent to the parents, and others phoned the parents to try and get oral consent if they had not replied when the vaccinations were due. Sometimes the parents did not fill in the consent forms properly, such as signing the form but not ticking yes or no. One school nurse in FG 2 said that she or he was happy if she or he even received the form back or got “some sort of signature.” She or he added that if the parents filled in the form wrong, she or he had to “phone them and explain the questions.”

This was most common in the non-Swedish-speaking families. The school nurses said that one positive point was that a signed consent form covered both doses of the HPV vaccine and further consent was not needed. All they had to do was to email the parents when the second vaccination was due, so that they were aware.

### Perception of Official Information Sources

The written information that was provided and the consent form the parents received were both taken from the county’s guidelines for school health services. The school nurses felt that the information was easy to understand and contained all the necessary information.

One school nurse from FG 2 said that she or he trusted the information she or he was being asked to pass on, and it was not “absolutely not” necessary to do her or his own research before every vaccination session. “Instead I presume that we should use what is in the guidelines, until something new comes along,” she or he said.

The school nurses said that the guidelines stated that they should send “something” from the Public Health Agency, but school nurses who were new to the role were not clear what that “something” was. Some of the school nurses said that they sent information from the Public Health Agency to the parents, together with the information letter and the consent form. The fact that the Public Health Agency also provided written information about HPV and the HPV vaccination in different languages was valuable for the school nurses when they needed to provide information for parents who did not speak Swedish.

Some school nurses felt that the information that the parents received about HPV should say that its aim is to protect their daughters against the HPV virus and that it should not be described only as a vaccine against cervical cancer. The reason for this is that it is a vaccine against certain types of HPV viruses that can cause various types of cancer, for example, cervical cancer, penile cancer, and also genital warts. They also said that the written information from the Public Health Agency regarding HPV and the HPV vaccination was trustworthy and that it provided the parents with support by providing standardized information material. This meant that the school nurses could rely on using the public health material instead of having to provide more personalized advice. The importance of who provided information was discussed, and the school nurses did not feel the pharmaceutical companies were trustworthy. Some felt that their goal was just to earn money, including a school nurse in FG 1, who said that “cash is king” about such companies. She meant that pharmaceuticals companies’ information was mainly written to sell more medicines, not to be so informative about the disease or the drug itself.

### Maintaining a Neutral Position on the HPV Vaccination

Most of the school nurses felt it was important to provide “neutral” information and not try to influence the girls and their parents. However, they told about an exception, a school nurse who communicated her or his positive opinions about the HPV vaccine and her or his experiences of vaccinating her or his own daughters. The school nurses expressed different opinions about whether they should tell the parents their own views or not. The general opinion was that they could only state that the official recommendation was that girls should take the vaccination. The most common attitude from the school nurses was that the HPV vaccination was being offered by the school health services, but it was up to the parents to decide whether their daughter should be vaccinated or not. It was not for the school nurses to persuade the parents to vaccinate their child, even if that was their professional view. The school nurses said that if they had more evidence about the benefits of the HPV vaccine, it would make it easier for them to emphasize the importance of the girls being vaccinated. One school nurse in FG 2 said school nurses wanted to “stress the importance of what you recommend” and added “I cannot say how it will look in 10–15 years, but this is how it looks today.”

Some school nurses said that they could continue to provide neutral information at the moment because there was already a high vaccination coverage among girls in Sweden. But if that situation would change and the coverage rates fell, then they would need to reevaluate what they said and how they should inform girls and their parents about HPV.

### Dealing With Dilemmas About Information and Conducting HPV Vaccinations

The school nurses described several dilemmas regarding HPV vaccinations. Sometimes the girl wanted to have the vaccination, but her parents refused, and this led to an ethical and moral dilemma. The school nurses said that their role was to encourage the girl to talk to her parents about why she wanted to get the HPV vaccination. However, it was ultimately the parents’ decisions, and even if the girls wanted the vaccine, the school nurses could not administer them without parental consent.

If the parents refused to vaccinate their daughter, some school nurses accepted the decision without further action, but others called the parents and invited them to a meeting to discuss HPV. Some parents made it clear that they did not want to discuss the vaccination, but others were more unsure about their decision, and this gave the school nurse the opportunity to discuss the issues in more detail.

The school nurses often met newly arrived immigrant families, with an interpreter, if they had not vaccinated their older girls against HPV. They talked to the girls and parents together. Many had not heard about HPV vaccination but were familiar with other vaccinations in the Swedish program. Most of them agreed to the vaccination, but sometimes they needed extra time or more information about the HPV vaccination and wanted to read translated material in peace and quiet before they decided.

The school nurses said that there may be other causes for a lower vaccination coverage than the parents’ lack of information. These included having an ethnic background and cultural views about sexuality. A common argument from parents with immigrant backgrounds was that the girls should not be sexually active until they got married, so the HPV vaccination was not relevant. Some school nurses said it was difficult to counteract those arguments.

They also said that targeting information to parents living in residential areas with several different nationalities was more difficult than reaching parents living in areas with predominantly Swedish families. As one school nurse said, “I find it quite difficult to reach parents with different nationalities and new arrivals. Families move all the time and I get the mail in return” (FG 3). The school nurses felt that consistency was important. One of the school nurses in FG 4 said that it was “very important” that everyone in the county should get the same information, and it should not depend on “which school nurse or school” was providing it.

According to the school nurses, the parents looked for information on the internet and could misinterpret what they found. They reported that in the child health care service, there is a routine stating that when parents decline any vaccination, they always will be booked for a meeting with the pediatrician for further discussion and explanation; they thought that it would be good if a school nurse in the same way could offer a meeting with the school doctor to explain the importance of HPV vaccinations. As a member of FG 3 said, “I hope the parents have enough information if they say no.”

According to the Public Health Agency, school nurses should continue to offer the HPV vaccination until the girls reach the age of 18 years, but it does not provide any information about how this should be done. A school nurse in FG 2 acknowledged the need to do this but said that the guidelines did not say how. She or he asked whether she or he was meant to “nag them” every year until they were 18?

### Building a Relationship and Trust With the Girls and Their Parents

The school nurses said that their relationships with the girls and their parents were important when it came to vaccination because this might help with any exchanges about the HPV vaccination. A school nurse from FG 1 said that some parents “had changed their minds” due to the existing relationships with the students, meaning that a bond of trust had been created, which made that the parents feel safe with the school nurses’ recommendation.

The school nurses also felt that it was essential that the girls trusted their school nurse. Going to an unknown facility that they did not have a relationship with would make it easier for them to refuse the HPV vaccination. It would also make it more difficult for the school nurse to motivate them and their parents to agree to the vaccination. The school nurses also stated that it was important for them to continue to have a good relationship with the family, even if the parents declined vaccination because they would have to deal with them on other school health matters.

### Making the Vaccination a Positive Experience for the Girls

The school nurses described the importance of making sure the actual vaccination was a positive experience. Their overall aim was to deliver it in a calm and secure atmosphere with no stress. There were always two school nurses participating in the vaccination, in accordance with the guidelines. The school nurse had the main responsibility, gave them the vaccination, and made sure that the girl felt secure, while the second school nurse documented the vaccination. The school nurses also wanted to make sure the girls felt prepared, as this would enhance their feeling of security. For example, school nurses planned carefully for the vaccinations to be given early in the day or that the teacher followed the girls to the school nurse in pairs, so that they did not have to wait and become nervous or stressed. They also encouraged the girls to eat breakfast and not come for their injection on an empty stomach. Parents were welcome to attend the vaccinations with their daughter if that helped.

The school nurses also said that the class teachers were very helpful when it came to organizing the girls into groups and making sure they came for their vaccinations in the right order. “The teachers were amazing and I got incredible support from them,” said a school nurse from FG 3. “There was good cooperation.”

## Discussion

The uptake of the HPV vaccination in Sweden is lower than desired. In 2018, it was 85% for the first dose and 80% for the second (Public Health Agency of Sweden). School nurses play an important role in reaching the target level and helping young girls and their parents to make informed decisions about the vaccination. Our results show that school nurses experienced challenges with regard to their role in HPV vaccinations, and the main issue was balancing a passive, neutral role and a more active approach. They strived to stay in a neutral position when it came to informing the girls and their parents about the Swedish recommendations regarding HPV and the HPV vaccination. They said that they wanted to preserve the autonomy of the girls, and this meant that they did not want to influence or persuade them or give them personalized advice (Fernbach, 2011). This created a dilemma. It was obvious that while they acknowledged the importance of high vaccination coverage, they did not see their role as increasing the uptake. Their role was to focus on giving the girls and their parents the information they needed to make up their own minds. This approach has also been reported in similar situations, with regard to prevention such as midwives’ views of their role in antenatal screening (Ahmed et al., 2013). The more passive role taken by medical staff may be a result of the stronger focus on patients’ autonomy (Krantz et al., 2004) and rights in today’s health care. However, the school nurses’ respect for the autonomy of the parents and girls did not exclude them from actively discussing the HPV vaccination, rather than just providing them with information (Romijnders et al., 2019). A study of parents’ views on information about HPV and the associated vaccination showed that they wanted to talk to the school nurse as well as receiving written information (Gottvall et al., 2013; Grandahl, Oscarsson, et al., 2014). It seems that the school nurses’ role with regard to HPV vaccinations needs to be further explored and defined. In addition, it was obvious in the results that the school nurses did not always base their actions in best practice and evidence. Previous studies have shown that recommendations from medical staff were one of the strongest predictors for the uptake of the HPV vaccine (Ames et al., 2017; Clark et al., 2016; Dorell et al., 2013; Gilkey & McRee, 2016; Rahman et al., 2015; Smith et al., 2017; Underwood et al., 2016; Wang et al., 2019; Ylitalo et al., 2013). The school nurses in our study said that they offered the HPV vaccine as an option, rather than strongly recommending it. Thereby, the opportunity to vaccinate individual girls could be missed and that could have contributed to the low national coverage to date.

The school nurses we spoke to found different ways to provide the girls and their parents with information about the HPV vaccine, with regard to the content and how it was delivered. These findings reflected another Swedish study, which also showed that the school nurses delivered information about the HPV vaccine to parents and girls in different ways. Most of the parents (97%) received written information, 59% of the school nurses gave the girls oral information, and only 17% provided information at parents’ meetings (Grandahl et al., 2017). The same variation in information routines was seen in an American study of pediatricians and pediatric nurses who provided the HPV vaccine citation. The amount and content of information varied substantially from just distributing standard information sheets and suggesting that the HPV vaccination was optional to discussing that it could prevent cancer (Garbutt et al., 2018).

The written information that the school nurses in our study distributed was taken from the county’s guidelines for school health services, and they sometimes included information from the Swedish Public Health Agency. The school nurses thought that the information they had been provided was adequate and trustworthy, but the variations in the way they used it indicated that the guidelines were not clear about how the information should be provided to the girls and their parents. Guidelines should offer concise instructions on how to provide health care services, and if they exist, they are likely to reduce inappropriate variations in practice (Graham & Harrison, 2005). In addition to guidelines, the school nurses have a responsibility to work based on prevailing evidence. This lack of knowledge regarding current evidence became obvious as some of them only relied on the county guidelines.

Our school nurses said that it was challenging when parents hesitated about, or refused to, vaccinating their child against HPV, and these situations required special strategies. Many of the school nurses contacted the parents to make sure they had adequate information. This was more proactive than the approach reported by Garbutt et al. (2018) where many medical staff abstained from further discussions with hesitant parents, as they saw these as time consuming. The school nurses also reported that they faced ethical and moral dilemmas when the girls wanted to be vaccinated, but their parents refused consent. They found this difficult because they couldn’t do so much to support the girls.

Providing HPV vaccinations to girls from newly arrived immigrant families was more challenging than if they were Swedish nationals, in terms of information and logistics. The school nurses felt there was a lack of support, or guidelines, on how to deal with situations that fell outside the box. Other studies have stressed that the importance of relevant and trustworthy information is even greater for immigrant parents, with regard to the HPV vaccination and vaccinations in general (Gilkey et al., 2016; Harmsen et al., 2015). Those studies also acknowledged that delivering this information was time consuming.

Altogether, our results indicate that offering the HPV vaccination to young girls can be a complex situation for school nurses. It can also cause the school nurses moral distress (Powell et al., 2018), for example, when the girl wants the vaccination but her parents refuse. The school nurse’s role in matters such as HPV vaccinations has been described as ambiguous and might lead to negative consequences for the individual school nurses with regard to the recommendations and dialogue they have with the girls and their parents (Powell et al., 2018).

### Strengths and Limitations

One Swedish county was selected to participate in the study, and it may not have represented other counties in Sweden. However, the participating school nurses represented different geographic and socioeconomic areas in the county. Because we recruited 17 of the 50 school nurses in the county, it is possible that the school nurses who took part had strong ideas about their experiences and attitudes and did not necessarily reflect the opinions of those who didn’t take part. The FG lasted between 45 and 80 min, mainly due to varying numbers of participants in each FG. Another strength was that the first FG was planned as a pilot, gave a rich data, and was included in the study.

### Implications for School Nursing

The findings from this study are of interest to school nurses and other providers of HPV vaccinations, who face the same dilemma of balancing a neutral position with the need to encourage HPV vaccination uptake. However, it should be pointed out that the school nurses in our study saw their role was helping the girls and parents make informed decisions about the HPV vaccine rather than increasing vaccination uptake. The information process tended to be rather complex, and the school nurses had to take a number of factors into account. We concluded that the school nurses needed clearer guidelines, a policy, and specific interventions to assist them when offering the HPV vaccination. There is also a need for continuous education, support, and evidence-based information to the school nurses. This would provide greater consistency and help school nurses to deal with difficult situations such as cultural beliefs and family disagreements.

It would also help them to have positive discussions with parents, which promote positive attitudes to the HPV vaccination and increase HPV vaccination uptake.

## Conclusion

The study highlighted the complex process that school nurses faced when they offered the HPV vaccination to girls. The school nurses balance between trying to keep a neutral position and not to influence, when they at the same time describe the importance of a good vaccination coverage. There were considerable variations in how the girls and their parents received written and oral information about the HPV vaccination, as confirmed in other studies. In order to increase vaccination coverage, it is important to provide school nurses with effective support and clear guidelines. This will enable them to play a more active role in interacting with the girls and their parents rather than just providing them with information.
